# CINNAMON-GUI: Revolutionizing Pap Smear Analysis with CNN-Based Digital Pathology Image Classification

**DOI:** 10.12688/f1000research.154455.1

**Published:** 2024-08-06

**Authors:** Luca Zammataro

**Affiliations:** 1 Lunan Foldomics LLC, Houston, TX, 77057, USA

**Keywords:** python, shiny, tensorflow, CNN, image-processing, digital-pathology, cervical-cancer, pap-smear

## Abstract

**Background:**

Medical imaging has seen significant advancements through machine learning, particularly convolutional neural networks (CNNs). These technologies have transformed the analysis of pathological images, enhancing the accuracy of diagnosing and classifying cellular anomalies. Digital pathology methodologies, including image analysis, have improved cervical cancer diagnostics. However, existing commercial platforms are often costly and restrictive, limiting customization and scalability.

**Methods:**

CINNAMON-GUI is an open-source digital pathology tool based on CNNs for classifying Pap smear images. Transitioning to a Shiny app in Python, it offers enhanced user interface and interactivity. The application supports dynamic web interactions, advanced features for image analysis, and state-of-the-art CNN models tailored for digital pathology. Key features include intuitive UI components, real-time image and plot generation, memory-efficient data handling, and robust training capabilities with customizable CNN architectures. The tool also integrates with Labelme for defining regions of interest and allows testing on external biospecimens.

**Results:**

Model A (seed 42, 100 epochs) and Model B (same architecture with adjusted augmentation parameters) were compared. Model A stabilized with training accuracy around 0.88 and validation accuracy around 0.913. Model B showed improved performance with training accuracy around 0.91 and validation accuracy around 0.95. Feature mapping highlighted critical morphological aspects, improving classification accuracy. Model B reduced misclassification errors significantly compared to Model A.

**Conclusions:**

CINNAMON-GUI demonstrates the potential of an open-source platform in digital pathology, providing transparency and collaborative opportunities. The tool enhances diagnostic accuracy through feature map analysis and optimized CNN training. Future development aims to extend its application to other cancer types, leveraging its dynamic and user-friendly interface for broader use in diagnostics.

## Introduction

Cervical cancer is a significant cause of death for women worldwide. In 2020, there were approximately 604,000 new cases and 342,000 deaths. ^1^ This type of cancer is closely linked to persistent infection by certain types of human papillomavirus (HPV), particularly HPV-16 and HPV-18, which account for about 70% of cervical cancer cases. ^2^^,^^3^ The Pap test, also known as the Papanicolaou test, is a widely used screening test for the prevention and early detection of cervical cancer. It involves examining cells collected from the cervix’s surface to identify any abnormal cellular changes indicative of potential cancer or precancerous lesions. ^4^ However, interpreting Pap test results can be subjective and reliant on the operator’s expertise. Additionally, the test’s accuracy can be affected by various factors, including sample quality and dyskeratotic cells, which can be challenging to interpret. ^5^

The field of medical imaging has witnessed significant advancements due to the integration of machine learning techniques, particularly convolutional neural networks (CNNs). ^6^ These technologies have transformed how pathological images are analyzed, enhancing accuracy in diagnosing and classifying cellular anomalies. Digital pathology methodologies based on image analysis have been developed to enhance the accuracy and effectiveness of cervical cancer diagnosis. ^7^^,^^8^ Existing commercial platforms, like Hologic’s ThinPrep.

Imaging System, BD’s FocalPoint GS Imaging System or Roche’s CINtec PLUS Cytology, integrate machine learning techniques to automate and refine the analysis of Pap smear tests, aiming to increase the speed and reliability of diagnostics. However, these technologies often come embedded within proprietary systems that are costly and restrictive in terms of customization and scalability.

In this paper, we present Cinnamon-GUI (Convolutional Neural Network and Multimodal Learning with a Graphic User Interface), an open-source tool that allows users to configure sophisticated CNN architectures for classifying digital biomedical images. The tool is particularly designed for the cervical Pap smear dataset Sipakmed, a significant resource in cervical cancer research due to its comprehensive collection of high-quality images and associated clinical data. ^9^

Cinnamon-GUI is a unique advancement in digital pathology, offering an open-source solution based on CNN for classifying Pap smear images. Its standout features include transparency, collaboration, and the ability for users to explore, modify, and tailor the code to their specific needs. Cinnamon-GUI is open-source approach not only invites the scientific community to actively contribute to its development but also fosters a sense of belonging and shared progress, enhancing its efficiency and applicability through feedback and updates.

## Methods

### Implementation

Cinnamon-GUI has transitioned to a Shiny app in Python, bringing several advantages, notably an enhanced user interface and improved interactivity. The Shiny framework ^10^ allows for dynamic and responsive web applications, making it easier for users to interact with machine learning models and visualize results in real-time.

The application stands out for its advanced features that optimize the analysis of digital images, making it a powerful tool for the scientific community. One of its main features is using state-of-the-art TensorFlow ^11^ convolutional neural networks (CNN) models which can recognize and classify complex patterns in pathological images, significantly improving diagnostic accuracy. Our implementation also makes extensive use of Python libraries such as Numpy, ^12^ Scipy, ^13^ Pandas, ^14^ and Scikit-learn. ^15^

**Figure 1.**
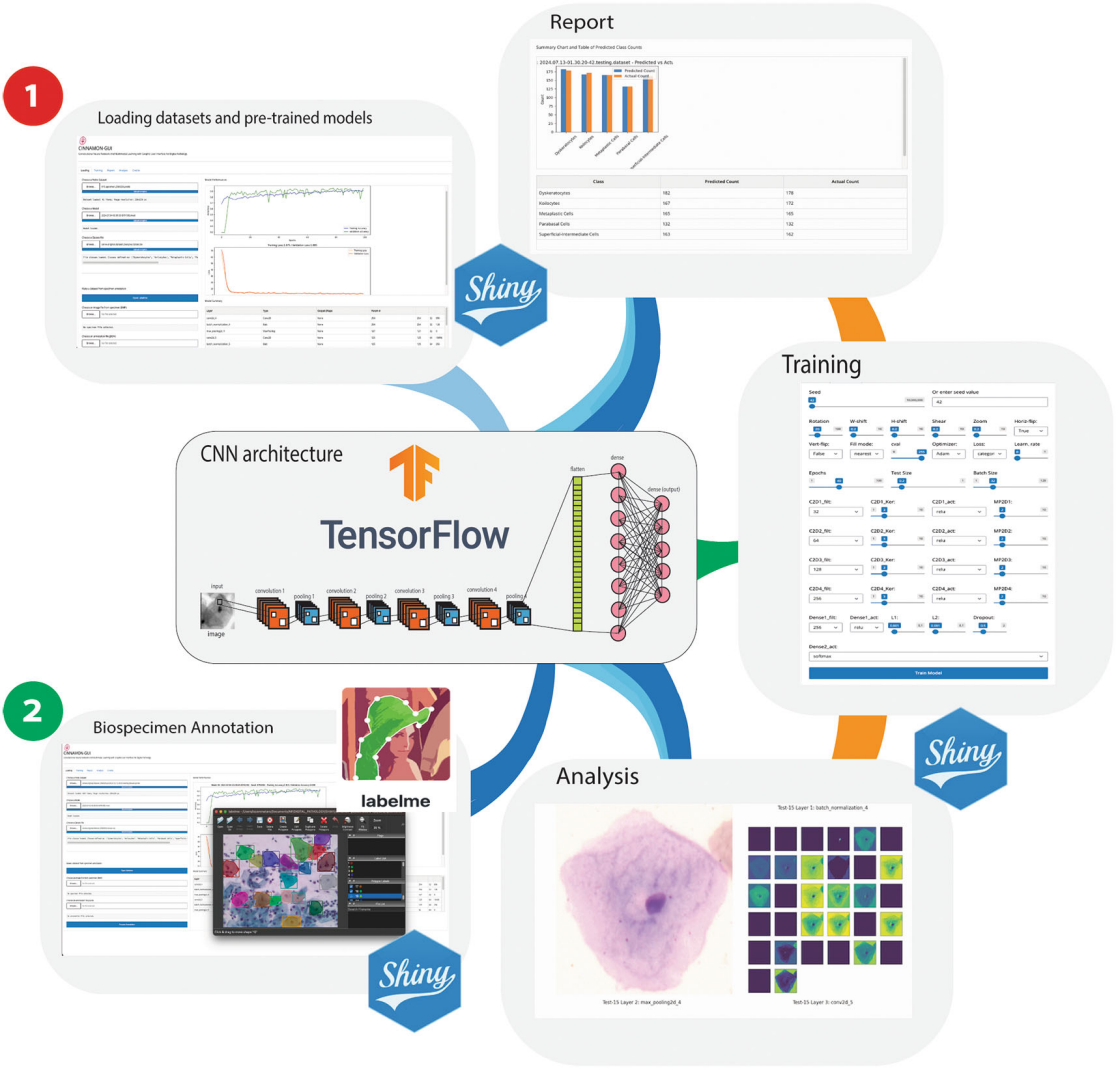
Overview of the dual workflow capabilities in Cinnamon-GUI. ** This schematic illustrates two primary operational pathways within the platform: 1) Data Processing and Model Training Workflow, where users can load pickle files from datasets to train models and generate reports post-testing, and 2) Biospecimen Annotation and Analysis Workflow, where users can load annotated Pap smear images, with annotations applied using the integrated Labelme software. This workflow supports loading the corresponding JSON annotation files and converting them and the images into a pickle dataset format for subsequent cell type prediction and classification. Additionally, this pathway facilitates the creation and expansion of datasets, enhancing the utility of Cinnamon-GUI for diverse research applications.

The general structure of the CNN in Cinnamon-GUI, characterized by convolutional layers, four pooling layers, one flatten layer and two dense layers, can be illustrated by Figure 1.

The user interface of Cinnamon-GUI is designed to be intuitive and dynamic, facilitating workflow even for professionals without advanced technical expertise. Dynamic UI components allow users to interact easily with machine learning models and visualize results in real time.

Another distinctive feature of Cinnamon-GUI is the dynamic generation and display of images and plots. Using base64 encoding, the application efficiently manages memory usage, ensuring high performance even when analyzing large datasets.

Upon startup, Cinnamon-GUI initializes a log file (log.txt) to record all activities and messages during the session. This logging system is integrated with a real-time notification system that informs users about the status and progress of various operations, such as model training, data augmentation, and image classification. This feature enhances transparency and traceability of the analysis process, keeping users informed about the ongoing tasks.

The application displays real-time progress during training, including a progress bar and status messages. The training process includes advanced features such as callbacks for early stopping and learning rate reduction based on validation loss. Early stopping prevents overfitting by stopping the training process when the model’s performance on a separate validation dataset stops improving. Learning rate reduction adjusts the learning rate during training to help the model converge to a better solution. These features significantly improve training efficiency. The trained model and log file are automatically saved to disk upon completion.

The Training Tab includes robust data augmentation capabilities that enhance the diversity of the training dataset and improve model generalization. Users can customize various augmentation parameters, including:

• Rotation Range: Randomly rotates images within a specified degree range.
• Width and Height Shift Range: Randomly shifts images horizontally and vertically.
• Shear Range: Applies shearing transformations.
• Zoom Range: Randomly zooms into images.
• Horizontal and Vertical Flip: Flips images horizontally and vertically.

These augmentation techniques are applied in real-time during training, generating new variations of the images in each epoch. ^16^

Finally, Cinnamon-GUI offers advanced tools for image display and feature mapping. Users can select an image index to display the image and its predicted class. Additionally, feature mapping plots can be generated to visualize the activations of different layers in the model. The application generates tests on the testing dataset to evaluate model performance, comparing predicted labels with actual labels and providing detailed reports.

The Cinnamon-GUI’s feature mapping function, which involves mapping the various layers of the CNN through our software, makes identifying these pathological traits much more accessible.

As example of implementation, we use the Sipakmed Dataset (see Data and Software Availability). Although almost all the models we experimented with had the architecture, we conducted several tests to understand which parameters were involved in improving classification performance. These performances were evaluated based on the number of failures obtained through the evaluation of the testing datasets, trying to identify which cells the various models failed to classify correctly.

We will discuss two comparative models, which we will call A (2024.07.13-01.30.20-42) and B (2024.07.01-00.58.38-42). Models A and B are identical in terms of their architecture, and optimization algorithms: both the Models use *Adam*, which combines the benefits of two other extensions of stochastic gradient descent, Adaptive Gradient Algorithm ( *AdaGrad*) and *Root Mean Square Propagation.*

However, the two models present some differences in the parameter settings for image augmentation. Both models were configured with a seed of 42, thus ensuring identical training sets for training and the reproducibility of tests using the same testing dataset. The latter consists of 809 images, equally distributed among the five categorical classes of Sipakmed.

*Model A*:

• Parameters: seed 42, 100 epochs, test size 0.2, batch size 32, rotation_range=20, width_shift_range=0.5, height_shift_range=0.4, shear_range=0.4, zoom_range=0.5.
• Performance:
  • Training accuracy: stabilizes around 0.88.
  • Validation accuracy: stabilizes around 0.913 with some fluctuations.
  • Training loss: stabilizes around 2.047.
  • Validation loss: stabilizes around 2.155.
  • Misclassification: 28 dyskeratotic cells misclassified as Koilocyte cells ( Figure 3).

*Model B*:

• Parameters: seed 42, 100 epochs, test size 0.2, batch size 32, rotation range=20, width_shift_range=0.2, height_shift_range=0.2, shear_range=0.2, zoom_range=0.2.
• Performance:
  • Training accuracy: stabilizes around 0.91.
  • Validation accuracy: stabilizes around 0.95 with more variability compared to training accuracy.
  • Training loss: stabilizes at 1.544.
  • Validation loss: stabilizes around 1.381.
  • Misclassification: Reduced total errors to 38, with dyskeratotic cell misclassifications down to 3 ( Figure 4).

The CNN architecture chosen for classifying the Sipakmed dataset is detailed in Table 1.

**Table 1.** The Cinnamon-GUI's architecture.

Layer (type)	Output shape	Param #
conv2d_1 (Conv2D)	(None, 254, 254, 32)	896
batch_normalization_1 (BatchNormalization)	(None, 254, 254, 32)	128
max_pooling2d_1 (MaxPooling2D)	(None, 127, 127, 32)	0
conv2d_2 (Conv2D)	(None, 125, 125, 64)	18496
batch_normalization_2 (BatchNormalization)	(None, 125, 125, 64)	256
max_pooling2d_2 (MaxPooling2D)	(None, 62, 62, 64)	0
conv2d_3 (Conv2D)	(None, 60, 60, 128)	73856
batch_normalization_3 (BatchNormalization)	(None, 60, 60, 128)	512
max_pooling2d_3 (MaxPooling2D)	(None, 30, 30, 128)	0
conv2d_4 (Conv2D)	(None, 28, 28, 256)	295168
batch_normalization_4 (BatchNormalization)	(None, 28, 28, 256)	1024
max_pooling2d_4 (MaxPooling2D)	(None, 14, 14, 256)	0
flatten_1 (Flatten)	(None, 50176)	0
dense_1 (Dense)	(None, 256)	12845312
dropout_1 (Dropout)	(None, 256)	0
dense_2 (Dense)	(None, 5)	1285

### Operation

**Figure 2.**
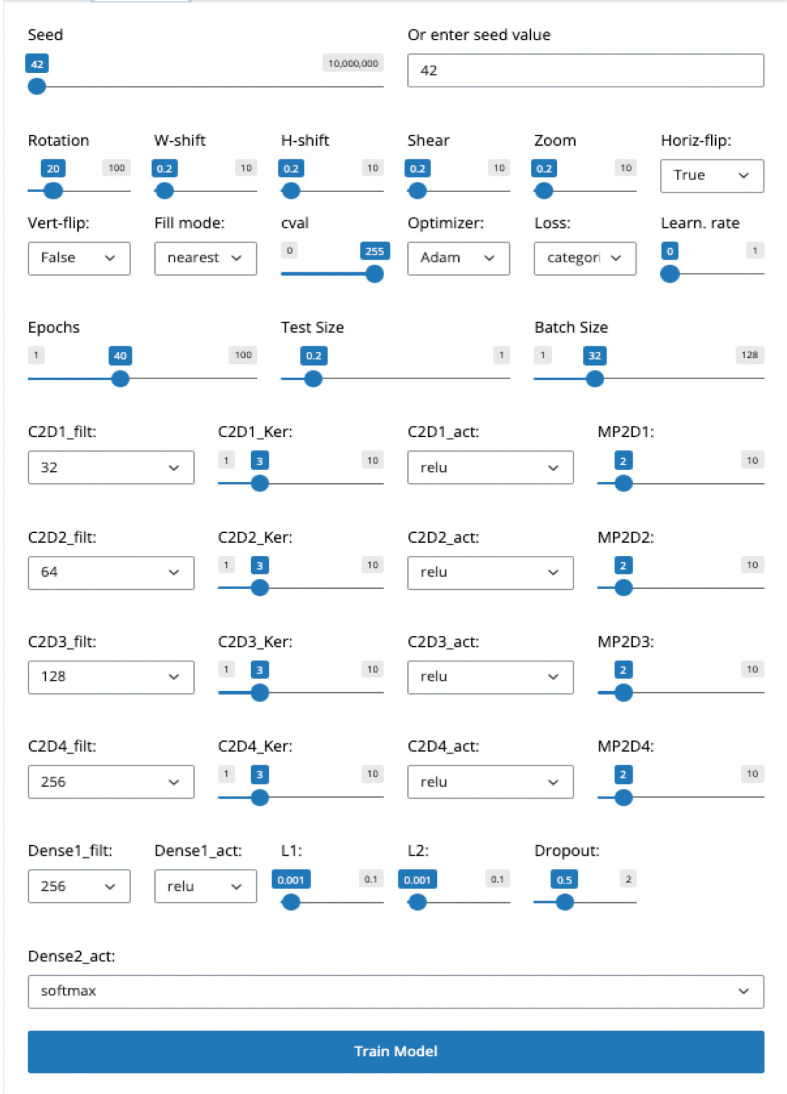
User interface for configuring model training parameters in Cinnamon-GUI. This interface allows users to adjust settings crucial for deep learning model training, such as seed value for reproducibility, data augmentation techniques (rotation, width and height shifts, shear, zoom, and horizontal flip), and other preprocessing options. Advanced settings include selecting optimizers, loss functions, and learning rate adjustments. Below, convolutional layer parameters such as filter count, kernel size, activation functions, and associated max-pooling layers are customizable along with dense layer configurations, regularization options (L1 and L2), and dropout rates, enabling precise control over the model's architecture and training dynamics.

1. Clone the Cinnamon-GUI GitHub repository:
git clone https://github.com/lunanfoldomics/Cinnamon-GUI

2. Create a virtual environment, and install required dependencies: follow the installation instructions from the GitHub repository
3. Navigate to the project directory:
cd Cinnamon-GUI/dashboard-tips
4. Start the application:
python cinnamon-gui.py
5. Access the application in your browser:
  Open your browser and navigate to ‘http://127.0.0.1:8000/’
6. Workflows:
  The Cinnamon-GUI platform offers two primary operational workflows. These workflows are designed to significantly enhance research efficiency and analytical accuracy in digital pathology studies, making the platform a valuable tool for researchers ( Figure 1).

1. **Data Processing and Model Training Workflow:** With its intuitive and user-friendly interface, this pathway empowers users to engage with machine learning. Users can easily import datasets stored as pickle files, a format that preserves the data structure, ensuring fidelity during loading. The workflow is designed around TensorFlow-based models, enabling users to train these models with their datasets or upload and use a pre-trained model.
  Once the dataset is loaded into memory, it is normalized, and reshaped for processing. Additionally, a class definition file (TSV format) can be uploaded to define the classes used in the dataset, facilitating labeling and classification during model training and evaluation.
  Users can configure and implement various customizable Convolutional Neural Network (CNN) architectures tailored to their specific needs through the Training Tab. The interface allows customization of multiple hyperparameters, including the number of convolutional layers, filters, kernel sizes, activation functions, and pooling layers. Users can add fully connected layers with customizable activation functions, dropout rates, and regularization techniques such as L1 and L2 ( Figure 2).
  Users can set various parameters for training the model, including number of epochs, batch size, learning rate, and data augmentation settings.
  The workflow includes internal functions for image normalization and a suite of functions for randomly splitting the dataset into training and testing sets for CNN learning. Users can select from a wide range of seeds for random splitting via the scikit-learn package using a dedicated sliding bar in the GUI’s Training Tab. Users can also choose from a variety of optimization algorithms to train their models.
  Optionally, users can upload a pre-trained model, the architecture of which is summarized and displayed in a tabular format. This feature provides detailed information about each layer of the loaded model and its parameters, helping users better understand the structure of the model.
  A significant feature of this pathway is its reporting capability; after training models and conducting tests, users can generate comprehensive reports. These reports visualize performance metrics such as accuracy, loss over epochs, and more detailed statistical analyses, providing crucial insights into model behavior and efficacy.
2. **Biospecimen Annotation and Analysis Workflow:** Tailored for researchers working with biomedical imagery, this workflow is crucial in dataset creation and expansion. It facilitates the loading and processing annotated Pap smear images in BMP format. Annotations are initially applied using Labelme, ^16^ a versatile tool that allows detailed marking of cells, essential for accurate specimen identification. The platform supports importing JSON files containing these annotations, which are then converted along with the images into pickle format. This data format standardization simplifies the integration of images into the machine learning pipeline, which is used for cell-type prediction and classification.
  When operating in Biospecimen Annotation and Analysis, Cinnamon-GUI can become a tool for diagnosing the possible presence of cancerous cells in the evaluated specimen.
  Furthermore, this workflow is instrumental in enabling researchers to build robust, annotated image datasets that enhance the utility of Cinnamon-GUI across various research applications.

## Results

**Figure 3.**
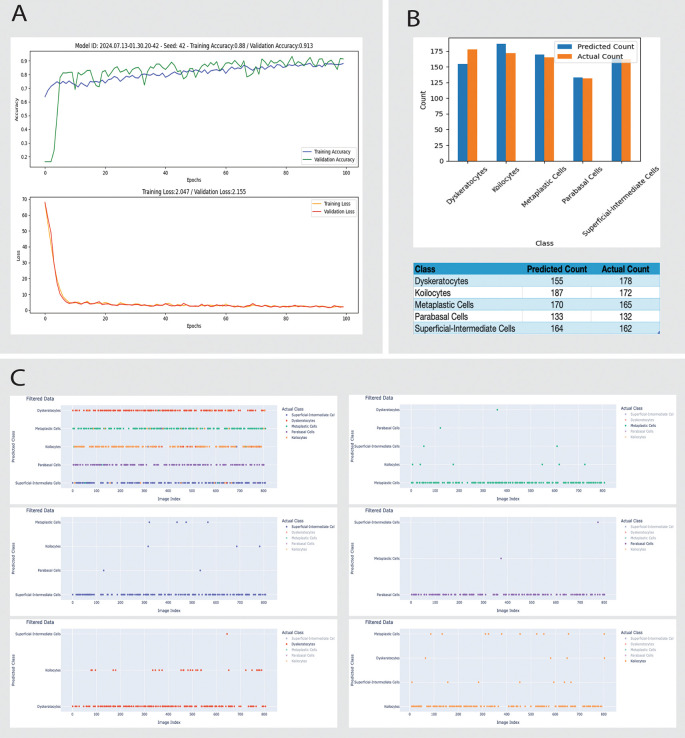
Comprehensive analysis of the machine learning Model A performance for classifying different types of cells in Pap smear tests using Cinnamon-GUI. A: Training dynamics over 100 epochs show training and validation accuracy and loss curves. The model achieves a training accuracy of 88.8% and a validation accuracy of 91.3%, with training and validation losses converging by the 100th epoch. B: Comparison of predicted versus actual counts of different cell types. The bar chart and accompanying table detail the count discrepancies across classes like Dyskeratocytes, Koilocytes, Metaplastic Cells, Parabasal Cells, and Superficial-Intermediate Cells, illustrating the model's predictive capabilities and areas for improvement. C: Scatter plot matrices represent the classification discrepancies for each cell type. Each plot filters data based on the predicted and actual class, highlighting specific images (by index) where discrepancies occur. This aids in visual error analysis and further model tuning.

The training accuracy for Model A increases rapidly in the initial epochs. Then, it stabilizes around 0.88, suggesting that the model learns from the training data but might not capture all the complexities. While slightly higher, the validation accuracy shows variability, indicating some instability. The training and validation losses for Model A indicate that the model is learning effectively but has room for improvement in generalization ( Figure 3).

A deeper analysis of model A’s feature maps and neuron activations shows that dyskeratosis and koilocytosis can have overlapping features, such as abnormal nuclear shapes and sizes. Model A might need to emphasize features like circular patterns and textures in both cell types. The highlighted regions in the feature maps show strong activations around these patterns (data not shown). There might be insufficient training examples for dyskeratoses in the Sipakmed dataset, causing the model not to learn the distinctive features adequately. For example, suppose data augmentation techniques introduce more prevalent patterns in koilocytosis. In that case, the model might learn to associate these patterns more strongly with koilocytosis, even when they appear in dyskeratoses.

**Figure 4.**
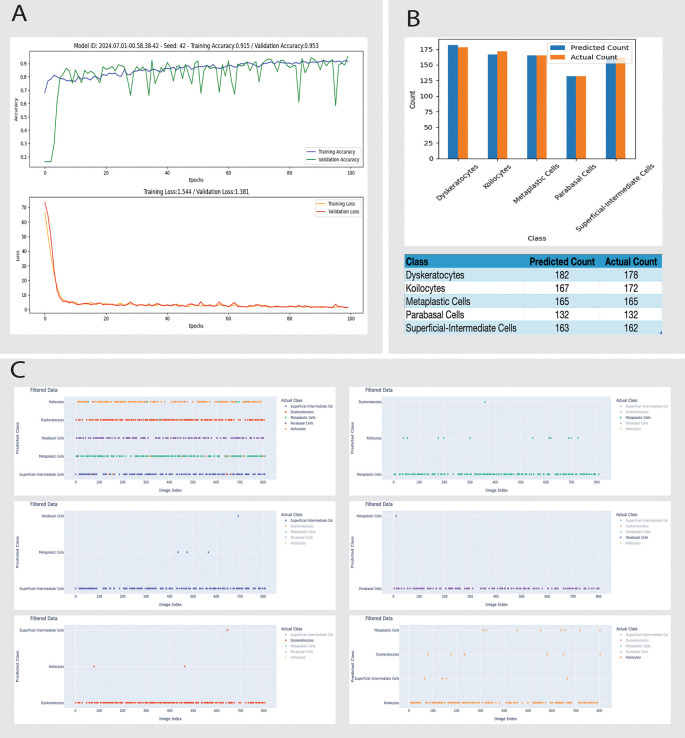
Detailed performance evaluation of the Model B for cell classification in Pap smear tests using Cinnamon-GUI. A: Training and validation performance over 100 epochs. The curves illustrate the model's training accuracy (top) stabilizing at 89.5% and validation accuracy (top) reaching 95.3%, with corresponding loss metrics (bottom) indicating good model convergence. B: Bar chart comparing the predicted versus actual counts for various cell types, including Dyskeratocytes, Koilocytes, Metaplastic Cells, Parabasal Cells, and Superficial-Intermediate Cells. The data demonstrate close alignment between predicted and actual counts, with a detailed breakdown in the accompanying table. Notably, the number of Dyskeratotic cells misclassified as Koilocytes has significantly decreased from 28 to 3, showcasing the model's improved specificity. C: Scatter plot matrices showing detailed discrepancies between predicted and actual classifications for each cell type. Each plot categorizes discrepancies by image index, allowing for targeted analysis of specific instances where the model's predictions deviate from actual classifications, facilitating focused diagnostic and model refinement efforts.

To improve CNN accuracy, we implemented the more balanced Model B, which demonstrates significant improvements. Reduced image augmentation parameters lead to better performance. The training and validation accuracies for Model B are higher than for Model A, and the losses are lower, indicating better generalization. The reduction in total errors and dyskeratotic cell misclassifications highlights the impact of optimized training data and model parameters ( Figure 4).

Using feature maps, we aim to discern the morphological aspects the CNN identified and utilized during training. Understanding these aspects allows us to isolate critical patterns, which could be fundamental for accurate classification criteria in pathology.

We will now present some observations derived from the feature mapping analyses. However, for editorial reasons, we will compare a superficial-intermediate cell, a koilocyte, and a dyskeratocyte from the Sipakmed dataset, classified using Model B. The feature maps can indicate that the CNN successfully captured various subcellular details, such as the nucleus’s structure and the cytoplasm’s distribution.

**Figure 5.**
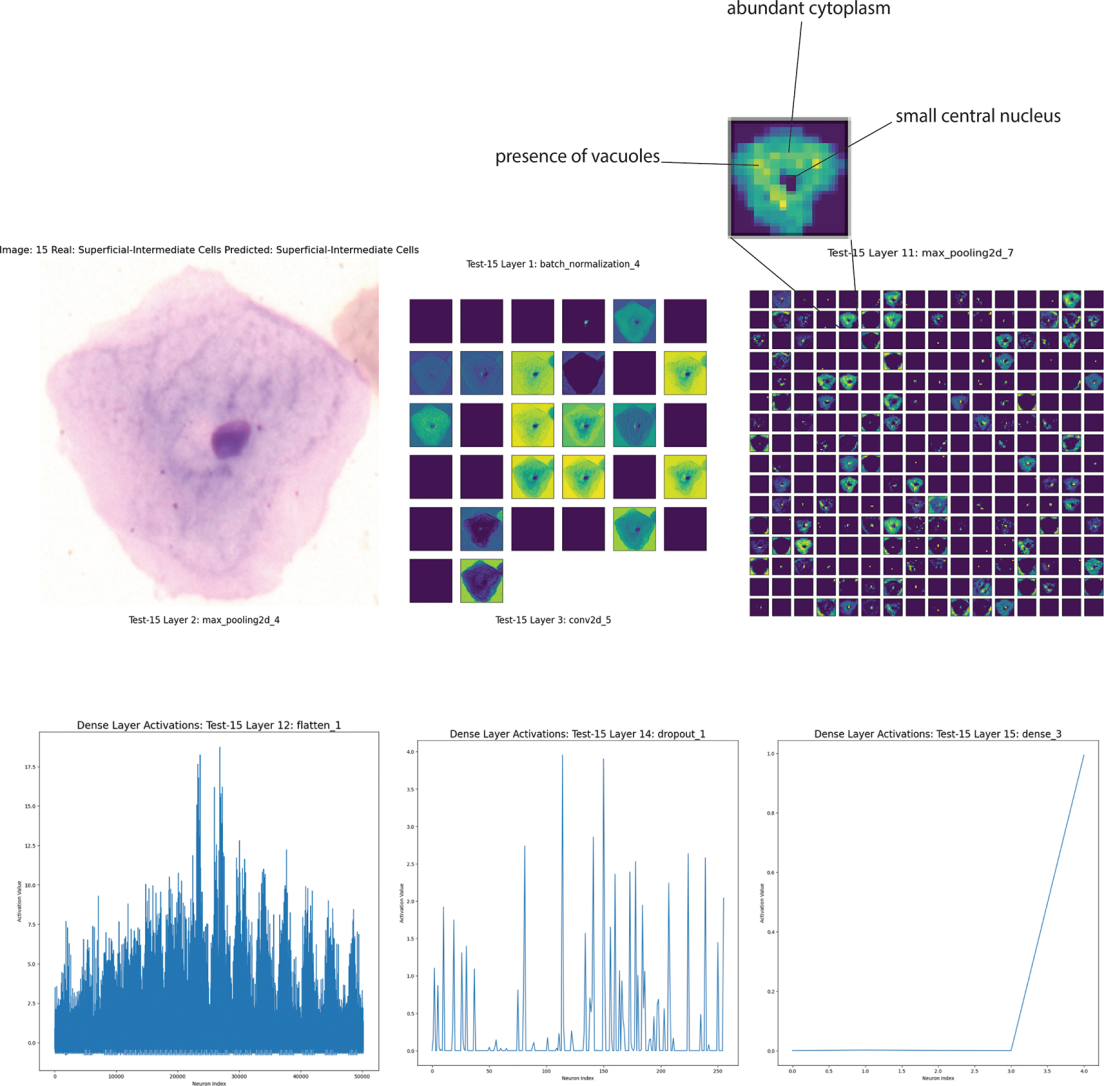
Analysis of a superficial intermediate epithelial cell from a Pap smear sample using Cinnamon-GUI. Top left: Original microscopy image of the cell classified as superficial intermediate due to its small central nucleus and abundant cytoplasm. Top right: Feature maps at various convolutional layers highlighting critical regions and texture variations. Bottom panels: Activation patterns in the dense layers demonstrate how neural network processing layers contribute to the final classification decision. The early layer visualizations emphasize the cell's contours and internal textures, while the deeper layers distill these features into a final diagnostic insight.

The cell in Figure 5 has been classified as a superficial intermediate, a type of epithelial cell frequently present in Pap smear samples. The cell has a relatively small central nucleus, while the cytoplasm appears abundant and evenly distributed. The feature map analysis of Layer 1 (Batch Normalization) plays a crucial role in distinguishing the cell’s contours, particularly the edges. This underscores the early layers’ ability to capture the essential characteristics of the cell’s shape and border. Layers 11 (Max Pooling) further emphasizes critical regions of the cytoplasm and nucleus, with reduced noise and better definition of subcellular features. The convolutions in this layer capture finer details, such as texture variations that could be related to chromatin structure or the presence of vacuoles.

The activations in the dense layers (flatten, Dense, Dropout) provide insight into the network’s decision-making process. They show how the network has condensed the information collected in the previous layers to make the final classification. High activations in certain regions indicate that specific features are essential for classifying as a superficial intermediate cell. ( Figure 5).

The feature mapping can distinguish dyskeratocytes from koilocytes, which are prodromal cells of a transformation into dyskeratocytes. Koilocytes (from the Greek, meaning “hollow vessel”) are squamous cells infected productively with episomal human papillomavirus (HPV) that exhibit morphological alterations indicative of HPV infection. Koilocytosis is pathognomonic, though not required, for diagnosing low-grade squamous intraepithelial lesions (LSIL). The main morphological alterations observed in koilocytosis include Karyolysis (chromatin within the nucleus appears fragmented and dispersed, giving the nucleus a translucent or granular appearance), demonstrating that the E5 and E6 proteins from HPV cooperate to induce koilocyte formation, hinting at nuclear alterations similar to Karyolysis. ^17^ Other alterations include nuclear hypertrophy (enlarged nucleus with increased chromatin density), nuclear enlargement, and hyperchromasia. ^18^ Koilocytes are indeed characterized by an increased nuclear/cytoplasmic ratio (the nucleus occupies more of the cellular space than the cytoplasm).

**Figure 6.**
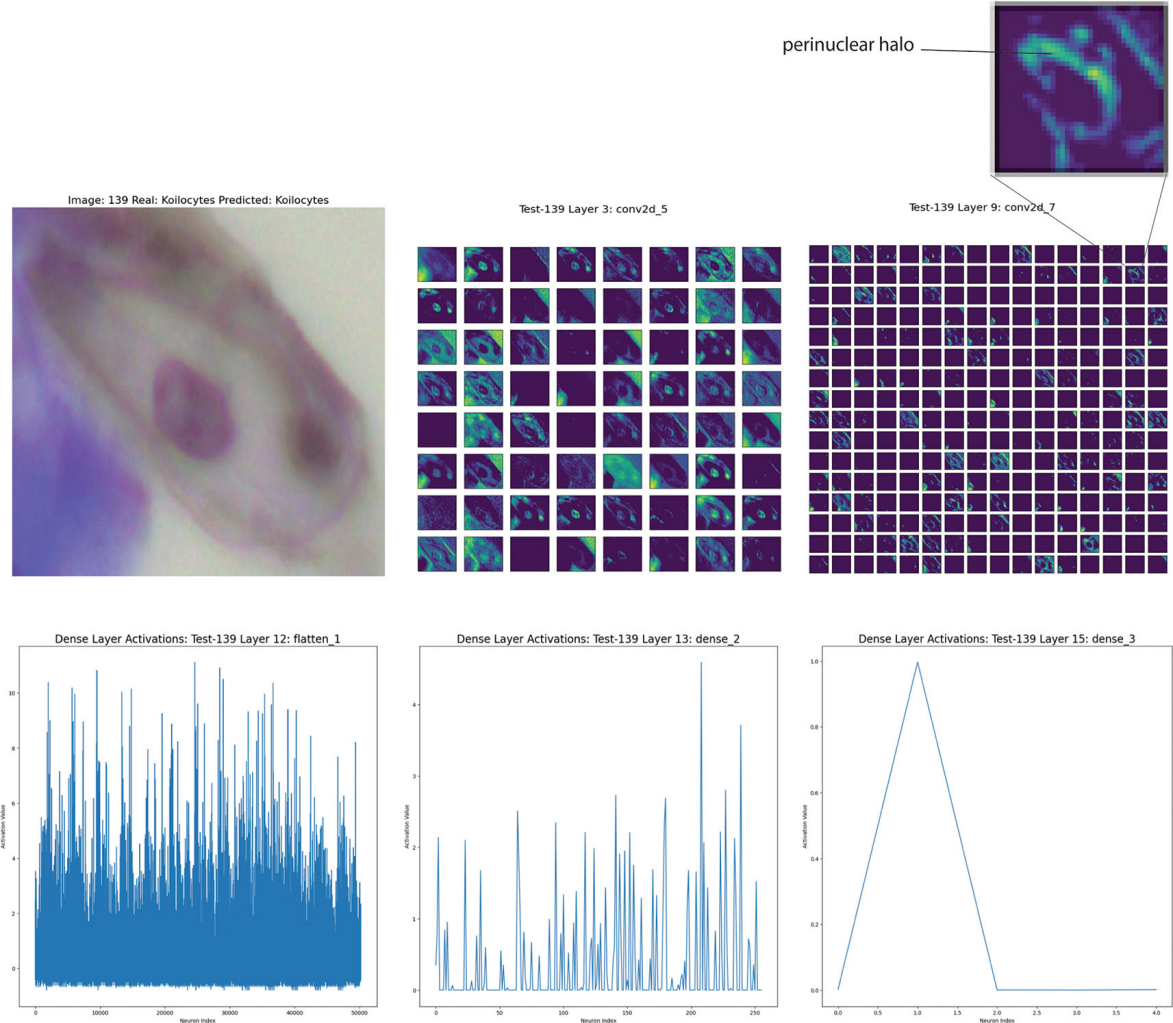
Detailed analysis of a koilocyte identified using Cinnamon-GUI. Top left: Original microscopy image showcasing the characteristic irregular nucleus with a clear central area and a denser perinuclear halo indicative of HPV effect. Top right: Close-up of the perinuclear halo emphasized in feature maps. Middle panels: Visualizations from Convolutional Layers 3 and 9 highlight the complex nuclear and cytoplasmic features, including the perinuclear halo and cytoplasmic vacuolations. Bottom panels: Neuronal activation patterns in the dense layers (flatten, dense layers 2 and 3).

The cell in Figure 6 represents a koilocyte correctly identified by Cinnamon-GUI; the nucleus is characteristically irregular and shows a clear central area with a denser peripheral zone known as the perinuclear halo. ^17^^,^^18^ This can be because of the HPV, which induces irregular peripheral collapse of keratins as an effect of the HPV’s E4 gene induction, leaving a clear space around the nucleus. ^19^

The feature maps in Layer 3 (Convolutional) show complexity, with a detailed representation of nuclear and cytoplasmic features. The perinuclear halo and nuclear irregularities are evident. Layer 9 (Convolutional) captures critical features, such as the perinuclear halo and cytoplasmic vacuolations. The CNN neuronal activations are highly specific to these patterns. As for the Superficial intermediate mentioned above, the final dense layers show a distribution of activations reflecting the model’s learning ( Figure 6).

**Figure 7.**
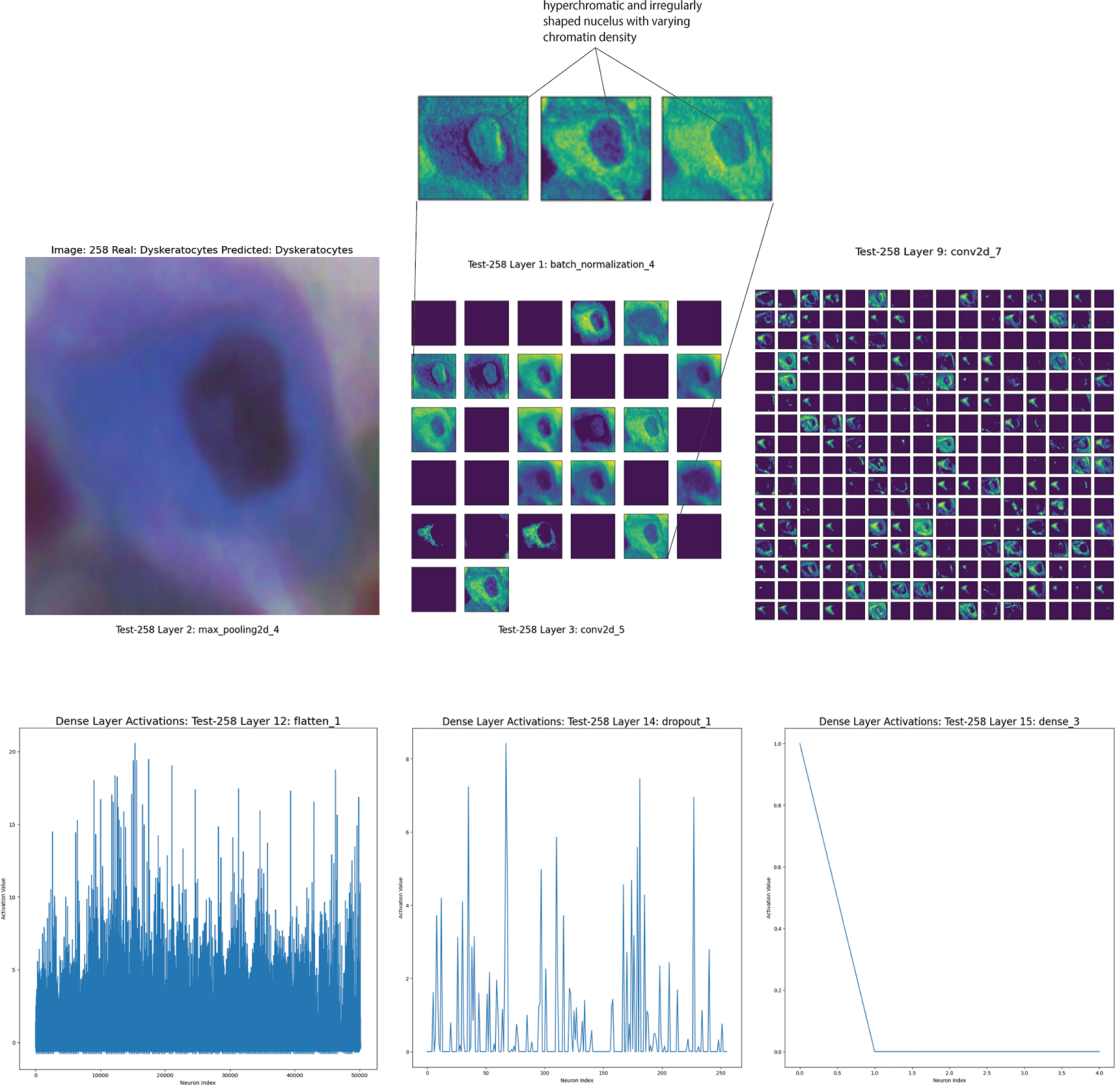
Analysis of a dyskeratotic cell using convolutional neural network techniques in Cinnamon-GUI. Top left: Microscopy image of the dyskeratotic cell displaying its characteristic irregular, dark, and compact nucleus alongside a dense and irregular cytoplasm with visible inclusions. Top right and middle: Feature maps from the convolutional and batch normalization layers highlight the cell's nuclear irregularities and cytoplasmic density variations, emphasizing the borders and internal textures. Bottom: Activation patterns from the dense layers demonstrate the CNN's ability to discern and focus on critical morphological features that help distinguish dyskeratotic cells from other cell types, showcasing intricate nuclear and cytoplasmic details crucial for accurate classification.

Finally, the image in Figure 7 shows a correctly identified dyskeratotic cell. The cell’s nucleus appears significantly altered with an irregular shape, and it is dark and compact. The cytoplasm is dense and irregular, often with evident inclusions. Dyskeratotic cells tend to show more intense and granular cytoplasm than normal cells.

The first layers (Batch Normalization and Max Pooling) show the detection of the cell’s basic contours. The patterns highlighted here focus on the cell’s borders and intensity differences in the cytoplasm.

The intermediate layers (Convolutional and Batch Normalization) focus more on nuclear features. The model highlights areas with intensity variations in the nucleus, hyperchromatic and irregularly shaped, with varying chromatin density, indicative of potential precancerous changes. The feature maps in the more advanced layers become more complex, showing intricate patterns that combine nuclear and cytoplasmic contours. These layers allow the model to distinguish dyskeratotic cells from other cell types ( Figure 7).

## Conclusions/Discussion

Although various machine learning-based solutions for digital pathology exist, the effort to build an utterly open-source platform for physicians and researchers can represent an additional diagnostic tool for the progress of digital pathology. Often, current software solutions compete, striving to propose increasingly accurate solutions for early cancer diagnosis. However, most of the time, it is necessary to deal with the limitations of available datasets. These limitations pose challenges even for the most sophisticated machine learning algorithms, not to mention the risk of overfitting on relatively small datasets, such as Sipakmed.

Conversely, Cinnamon-GUI proves to be an extremely transparent tool that aims to identify the real problems of the used datasets through the analysis of feature maps, alerting researchers about the quality of predictions. Through our experience, we have, for example, understood that more issues related to inaccurate classification than the chosen CNN architecture could depend on how we attempt to extend the original dataset through image augmentation. We have realized that this is an essential part of modern machine learning and that these techniques must be used cautiously.

The analysis of the feature maps and neuron activations suggests that if not accurately trained, the CNN model can confuse specific patterns present in both dyskeratoses and superficial-intermediate cells. To improve classification accuracy, it is crucial to strengthen training by playing with image augmentation settings and refining the model architecture to capture distinctive features better.

We have demonstrated how image augmentation can benefit a CNN model’s performance improvement when applied in “small doses” and that it may not be necessary to hyper-transform the original dataset. Instead, reducing parameters such as width and height shift can yield better results. Indeed, using Model B, we decreased the number of errors significantly, demonstrating the critical impact of fine-tuning augmentation settings on model performance and accuracy.

With the help of feature mapping, we understand the role of the early layers of the network, which are particularly effective in reinforcing contours and identifying the general shape of the cell. The subsequent and dense layers can capture fine details and texture differences crucial for accurate classification. The activation peaks indicate the most significant features the model used to make the classification.

Cinnamon-GUI, with its fully interactive interface, controls, and extreme ease of use, along with its accurate plots, represents a user-friendly tool in the field of Digital Pathology. It allows even non-experts to manipulate complex CNN architectures without any programming experience, empowering them to contribute to the field. Although the CNN models implemented within the code are limited to four convolutional layers and four pooling layers, the number of configurations that can be explored with our tool is vast, giving users confidence in their ability to explore and experiment.

Another powerful feature of Cinnamon-GUI is the ability to import external specimens and build new datasets using a manual annotation system based on the LabelMe software.

Cinnamon-GUI can extend its training capabilities using external datasets provided by the user or be limited to making the best possible predictions and classifications when used as a query system.

Another necessary functionality is multimodal learning, which integrates various data types to enhance analysis accuracy. Although this capability is intended only for research purposes, it represents a significant step towards a more comprehensive understanding of pathological conditions through analyzing heterogeneous data. We believe that the use of Cinnamon-GUI is not limited to the analysis of Pap smears alone. We are working towards making Cinnamon-GUI a powerful platform for the early diagnosis of many other types of cancers in the future, instilling hope and optimism in the potential impact of our work.

## Data Availability

• Source code available from: https://github.com/lunanfoldomics/Cinnamon-GUI

• Archived software: https://doi.org/10.5281/zenodo.13109219
• License: OSI approved open license software is under GNU Affero General Public License v3.0. SPDX-License-Identifier: AGPL-3.0-only The Sipakmed database, ^9^ which consists of 4049 color images of cells from cervical pap smears, represents a vital example of this tool. Images have been classified into five cellular subclasses: Superficial-Intermediate Cells, Parabasal Cells, Metaplastic Cells, Koilocytes, and Dyskeratocytes. For our work, the database was restructured into a numpy array and subsequently inserted into a Pandas DataFrame, with each row corresponding to a sequence of 65536 pixels, each represented by an RGB triplet for color and associated with an output label. Once loaded into a NumPy vector, the images are reshaped into 256×256 matrices. Once the Sipakmed dataset is downloaded, it needs to be unzipped into a directory, which we might call “sipakmed.” The main directory structure of SipakMed is not particularly complex, but it is essential to understand where the images are located within the five cellular categories to correctly construct the pickle file. Therefore, a script must be generated to search for images within the sipakmed directory and generate the pickle file. A pickle version of Sipakmed is downloadable from here: https://www.kaggle.com/datasets/lucazammataro/sipakmed-dataset-for-cinnamon-gui The pre-trained Model B described in the paper is downloadable from here: https://www.kaggle.com/datasets/lucazammataro/cinnamon-gui-model-b-sipakmed/data You can create a Pickle Training Dataset from Sipakmed, downloading it from https://www.cs.uoi.gr/~marina/sipakmed.html
