# Supplementary material for: CINNAMON-GUI: Revolutionizing Pap Smear Analysis with CNN-Based Digital Pathology Image Classification
Source: F1000Res. 2024 Aug 6;13:897. [Version 1] doi: 10.12688/f1000research.154455.1 (PMC11801405; doi:10.12688/f1000research.154455.1)
Supplement: Peer review history

# Reviewer report: Reviewer response for version 1

**DOI:** 10.5256/f1000research.169489.r349037

**Published:** 2025-01-02

**Authors:** Gabriella Casalino

**Affiliations:** 1 University of Bari, Bari, Italy

The article "CINNAMON-GUI: Revolutionizing Pap Smear Analysis with CNN-Based Digital Pathology Image Classification" by Luca Zammataro presents an innovative open-source tool to improve the classification and diagnosis of Pap smear images. CINNAMON-GUI uses convolutional neural networks (CNNs) to address challenges in digital pathology, particularly in cervical cancer diagnostics. It combines sophisticated machine learning techniques with an interactive, user-friendly interface to make complex image processing accessible even to non-experts.

 One of the key strengths of the work is its open-source nature, fostering collaboration and customization within the medical and scientific communities. CINNAMON-GUI offers real-time interactivity, allowing users to visualize and understand classification results and analyze CNN layers to gain deeper insights into image features. The application is designed to support non-expert users while addressing a critical medical challenge, enhancing trustworthiness through transparency and adaptability. The capability to annotate, analyze, and integrate various datasets makes it a highly versatile tool. Including dynamic UI components and integrating with external software like Labelme further enriches its usability.

 Although the interface assumes some familiarity with the structure of deep neural networks, a potential limitation is its reliance on users' technical knowledge for customization. An additional layer of abstraction that provides default settings for absolute beginners could enhance accessibility. This would allow users with no technical expertise to benefit from the tool while gradually understanding its capabilities.

 Overall, this work is valuable to digital pathology and medical image analysis. Its potential to support the diagnosis of other pathologies in the future, coupled with the prospect of natural language explanations for identified regions, holds promise for broader adoption and impact. By prioritizing transparency, collaboration, and ease of use, CINNAMON-GUI is well-positioned to drive medical diagnostics and research advancements.

Are the conclusions about the tool and its performance adequately supported by the findings presented in the article?

Yes

Is the rationale for developing the new software tool clearly explained?

Yes

Is the description of the software tool technically sound?

Yes

Are sufficient details of the code, methods and analysis (if applicable) provided to allow replication of the software development and its use by others?

Yes

Is sufficient information provided to allow interpretation of the expected output datasets and any results generated using the tool?

Yes

Reviewer Expertise:

I work in computational intelligence, specializing in methods for intelligent analysis. My primary focus lies in the medical domain, where I have experience with a diverse range of medical data, including omics, acoustic features, vital parameters, video, and signal processing.

I confirm that I have read this submission and believe that I have an appropriate level of expertise to confirm that it is of an acceptable scientific standard.

# Reviewer report: Reviewer response for version 1

**DOI:** 10.5256/f1000research.169489.r312178

**Published:** 2024-10-09

**Authors:** Fabio Grizzi

**Affiliations:** 1 Humanitas University, IRCCS Humanitas Research Hospital, Rozzano, Italy

Luca Zammataro's article marks a significant advancement in the adoption of appropriate tools for analyzing cytological and histopathological entities, which are often challenging to assess objectively through microscopy alone. The proposed tool, Cinnamon-GUI (CINNAMON-GUI), highlights the potential of an open-source platform in digital pathology, offering both transparency and collaborative opportunities. This tool improves diagnostic accuracy by utilizing feature map analysis and optimized CNN training. Future developments aim to expand its application to other cancer types, broadening its role in diagnostics through its dynamic and user-friendly interface. While Zammataro introduces the tool for classifying Pap smear images, its underlying principles can be applied to other clinical challenges. This manuscript is highly recommended for researchers focused on computer-aided image analysis systems in their work.

Are the conclusions about the tool and its performance adequately supported by the findings presented in the article?

Yes

Is the rationale for developing the new software tool clearly explained?

Yes

Is the description of the software tool technically sound?

Yes

Are sufficient details of the code, methods and analysis (if applicable) provided to allow replication of the software development and its use by others?

Yes

Is sufficient information provided to allow interpretation of the expected output datasets and any results generated using the tool?

Yes

Reviewer Expertise:

My research focuses on tumor complexity and the interactions between tumor cells and both the innate and adaptive immune systems. This includes exploring non-linearity, chaos theory, fractals, and AI-based computer algorithms within the biological context.

I confirm that I have read this submission and believe that I have an appropriate level of expertise to confirm that it is of an acceptable scientific standard.
